# Role of ClpX and MsrA/B in the Oxidative and Cell Wall Stress Response in *Bacillus anthracis* Sterne

**DOI:** 10.1002/mbo3.70377

**Published:** 2026-08-16

**Authors:** Aeron B. Pennington, Salina Hona, Josey I. Austin, Kelsey C. Waite, Mikaela D. Stewart, Shauna M. McGillivray

**Affiliations:** ^1^ Department of Biology Texas Christian University Fort Worth Texas USA

**Keywords:** *Bacillus anthracis*, ClpX, methionine sulfoxide reductase, MsrA/B, oxidative stress, penicillin resistance

## Abstract

ClpX functions as a component of the ClpXP protease, a conserved intracellular protease that regulates protein turnover, stress responses, and virulence in multiple bacterial species. Our lab has established that *clpX* is necessary for resistance to cell envelope targeting antibiotics, such as penicillin and daptomycin in *Bacillus anthracis* Sterne. Previous microarray data identified the *msrA/B* gene encoding a bifunctional methionine sulfoxide reductase as upregulated in the Δ*clpX* mutant. Methionine sulfoxide reductases (Msr) repair oxidatively damaged proteins by reducing methionine sulfoxide residues back to methionine. While Msr enzymes are primarily associated with oxidative stress, cell wall antibiotics induce expression of *msrA1* and *msrB* in *S. aureus*. Here, we investigated the role of MsrA/B in oxidative and cell envelope stress. Our results show that although hydrogen peroxide and paraquat induce *msrA/B* expression, the Δ*msrA/B* strain was not susceptible to either oxidant, whereas the Δ*clpX* strain was sensitive to both. We also found that loss of *msrA/B* conferred penicillin‐specific sensitivity, but, unlike Δ*clpX*, increased sensitivity was not seen with other cell wall or cell membrane targeting antibiotics. Inactivation of the catalytic cysteine of either Msr domain of MsrA/B failed to complement, suggesting that the reducing activity of MsrA/B is required for penicillin resistance. These findings indicate that while MsrA/B contributes to penicillin resistance, other proteins in the ClpXP modulon must also play a role in oxidative and cell envelope stress.

## Graphical abstract

Methionine sulfoxide reductase (*msrA/B*) is an oxidative response enzyme that reduces oxidized methionine residues to maintain protein function. Surprisingly, loss of MsrA/B does not increase sensitivity to hydrogen peroxide or paraquat‐induced oxidative stress in *Bacillus anthracis* Sterne. Rather, MsrA/B is required for penicillin resistance but not for other cell wall targeting antibiotics. Penicillin resistance depends on the catalytic activity of MsrA/B, suggesting that its reducing activity may contribute to resistance by mitigating oxidative stress generated during penicillin treatment.

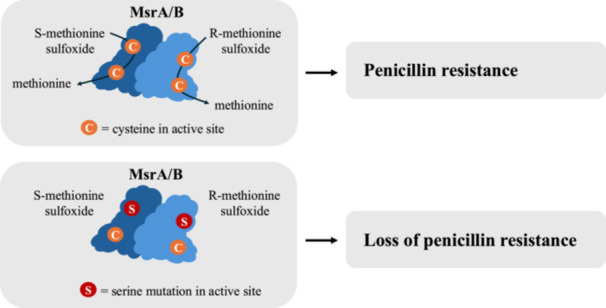

## 1 Introduction

Caseinolytic proteases (Clp) are major intracellular proteases that regulate protein turnover by degrading transcriptional regulators, metabolic enzymes, and damaged or nonfunctional proteins (Baker and Sauer 2012; Feng et al. 2012; Illigmann et al. 2021). These proteases are comprised of two proteins: a regulatory ATPase, such as ClpX, which recognizes and unfolds proteins, then feeds them to the proteolytic subunit, ClpP (Baker and Sauer 2012). Orthologs of the ClpXP protease are found in most bacterial species and regulate stress responses to heat, DNA damage, cell wall damage, and oxidative stress through the degradation of regulatory proteins, such as transcription factors, or damaged proteins (Frees et al. 2007; Illigmann et al. 2021; Aljghami et al. 2022). Loss of ClpX or ClpP also impairs virulence in several pathogens, including *Staphylococcus aureus, Listeria monocytogenes, Streptococcus pneumoniae*, and *Bacillus anthracis* (Frees et al. 2014; Gaillot et al. 2000; Robertson et al. 2002; Mebus et al. 2025; McGillivray et al. 2009).

Previous work has shown that *clpX* is critical for resistance to cell envelope targeting antimicrobials, such as penicillin and daptomycin, as well as the cathelicidin family of antimicrobial peptides (AMPs) in the Gram‐positive pathogen *B. anthracis* (McGillivray et al. 2009, 2012), the causative agent of anthrax; however, the mechanism behind this is unclear. To better understand why loss of ClpX results in these phenotypes, we compared gene expression between wild‐type (WT) *B. anthracis* Sterne and an isogenic Δ*clpX* mutant (Claunch et al. 2018). In total, 119 genes were dysregulated upon loss of *clpX*, including genes with connections to cell wall targeting antibiotics in other bacterial species (Claunch et al. 2018). One of these, the *lrgAB* operon, contributed to daptomycin and LL‐37 resistance in *B. anthracis* Sterne but, unlike *clpX*, loss of *lrgAB* had no effect on penicillin susceptibility (Claunch et al. 2018). This suggests that additional proteins play a role in mediating the ClpX‐associated response to cell envelope antimicrobials. Here, we focus on a gene upregulated in Δ*clpX*, the bifunctional methionine sulfoxide reductase gene *msrA/B*, originally annotated as *msrA* (Claunch et al. 2018), which has been linked to cell wall stress in *S. aureus* (Wilkinson et al. 2001; Singh et al. 2015).

MsrA and MsrB enzymes serve an essential role in the bacterial oxidative stress response by repairing proteins damaged by reactive oxygen species (ROS) (Stadtman et al. 2002; Ezraty et al. 2005). Upon exposure to ROS, methionine residues can be oxidized into methionine sulfoxide (MetO), impairing their function (Ezraty et al. 2005, 2017). There are two diastereomeric forms of MetO: S‐MetO and R‐MetO, which are reduced by MsrA and MsrB, respectively (Boschi‐Muller et al. 2005). Both enzymes share a similar mechanism for reducing MetO, with each possessing a catalytic site containing an essential cysteine residue that reduces MetO and a second, auxiliary cysteine that facilitates the regeneration of the catalytic cysteine, referred to as the recycling cysteine (Boschi‐Muller et al. 2005). In many bacterial species, deletion of *msrA* and *msrB* is associated with increased sensitivity to oxidative stressors, such as hydrogen peroxide (H_2_O_2_) and superoxide generators, such as paraquat, although the specific ROS‐sensitivity is variable (Ezraty et al. 2005; Sasindran et al. 2007; Singh et al. 2018). In some pathogens, including *Salmonella enterica* serovar Typhimurium, *S. aureus*, *S. pneumoniae*, *Enterococcus faecalis*, and *Francisella tularensis*, loss of either *msrA* or *msrB* decreases survival in macrophages and/or animal models of infection (Sinha et al. 2026; Singh et al. 2015; Saleh et al. 2013; Zhao et al. 2010; Saha et al. 2017).

Most bacterial species have at least one copy of MsrA and MsrB, though some possess multiple copies of the enzymes (Ezraty et al. 2005; Sasindran et al. 2007; Delaye et al. 2007). Although MsrA and MsrB are often independent proteins, a fused MsrA/B enzyme has been identified in several bacterial species, including *Neisseria* spp., *Streptococcus pneumoniae*, *Helicobacter pylori*, *Haemophilus influenzae*, *Fusiform nucleatum*, and *Bacillus cereus* (Skaar et al. 2002; Kim et al. 2009; Alamuri and Maier 2004; Nasreen et al. 2020; Scheible et al. 2022; Madeira et al. 2017). While independent Msr proteins are generally cytoplasmic (Aussel and Ezraty 2021; Singh et al. 2015; Andrieu et al. 2020), the cellular location of the fused MsrA/B is more variable. Some MsrA/B proteins are associated with the cell membrane, as seen in *Neisseria* spp., *H. pylori*, and *H. influenzae* (Skaar et al. 2002; Alamuri and Maier 2004; Nasreen et al. 2020), while others are predicted to be entirely cytoplasmic, as with the MsrA/B of *B. cereus* (Madeira et al. 2017). Further, *S. pneumoniae* and *F. nucleatum* possess both an intracellular MsrA/B and a second MsrA/B anchored to the cell membrane (Saleh et al. 2013; McGregor and Wolthers 2025). Some fused proteins additionally contain a thioredoxin domain, as seen in *Neisseria* spp. (Wu et al. 2005; Lowther et al. 2002) and the MsrA/B1 of *F. nucleatum* (McGregor and Wolthers 2025).

Although MsrA and MsrB have been associated nearly exclusively with oxidative stressors, research with *S. aureus* has linked the cell wall stress response and Msr enzymes. *S. aureus* has three copies of the *msrA* gene and one *msrB* gene, with *msrA1B* found as an operon while *msrA2* and *msrA3* are encoded separately (Singh and Singh 2012). Studies examining the cell wall stress response of *S. aureus* found elevated expression of *msrA1* in response to oxacillin, bacitracin, and d‐cycloserine (Wilkinson et al. 2001; Utaida et al. 2003). Despite induction of *mrsA1* by cell wall active antibiotics, deletion of the three *msrA* genes, individually or in combination, had no effect on antibiotic susceptibility, although it did increase sensitivity to ROS (Singh et al. 2015). Interestingly, loss of *msrB* in *S. aureus* increased resistance to cell wall antibiotics, suggesting that MsrB serves some function in this stress response (Singh et al. 2015). From this, we hypothesized that altering *msrA/B* levels in *B. anthracis* Sterne would change antibiotic susceptibility to cell wall active agents, with loss of *msrA/B* increasing resistance, as was seen with Δ*msrB* in *S. aureus*, and higher *msrA/B* levels which occur in Δ*clpX*, decreasing resistance. Contrary to our hypothesis, we found that loss of *msrA/B* increased sensitivity to penicillin but had no effect on susceptibility to other cell envelope targeting antimicrobials nor reactive oxygen species.

## 2 Materials and Methods

### 2.1 Bacterial Strains and Reagents

*Bacillus anthracis* Sterne strain 34F2 (pX01^+^, pX02^−^) was grown in brain‐heart infusion (BHI) media (Hardy Diagnostics) at 37°C under aerobic conditions, unless otherwise stated. The generation of the *B. anthracis* Sterne Δ*clpX* mutant and the *clpX* complementation strains using the plasmid pUTE657 has been previously described (McGillivray et al. 2009; Claunch et al. 2018). The following antibiotics were used for selection: *B. anthracis* mutants: 5 μg/mL erythromycin (Erm5), 100 μg/mL spectinomycin (Spec100); *E. coli*: 500 μg/mL (Erm500) and 100 μg/mL ampicillin (Amp100). All chemicals came from Sigma‐Aldrich, and all enzymes from NEB unless otherwise indicated. Mutant and complementation strains constructed for this study are described below. Primers used in this study are listed in Table 1. Gene locus tags are from the Ames Ancestor genome, but nucleotide sequences are identical to the Sterne strain.

**Table 1:** Primers used in this study.

Primer name	Sequence
pUTE657 fwd	5′‐GAACGTTGCTCGAGGGTAAATG‐3′
pUTE657 rev	5′‐GGTACGTACGATCTTTCAGCC‐3′
Gene specific primer‐1	5′‐ACGTATTTCCAGACGGTCCAGG‐3′
A primer fwd	5′‐GAAATGGAGAAGGACGTTAGAACA‐3′
A primer rev	5′‐CTGCCTCATAATGTCCCGTC‐3′
B primer fwd	5′‐CGACGGGACATTATGAGGCA‐3′
B primer rev	5′‐CTGGACCGTCTGGAAATACGTG‐3′
*ABC‐transporter* qPCR fwd	5′‐CACCTGGTCGCACAATTCGT‐3′
*ABC‐transporter* qPCR rev	5′‐TCTTCTGGTGTAGCAGTTCC‐3′
16S rRNA qPCR fwd	5′‐CACACTGGGACTGAGACACG‐3′
16S rRNA qPCR rev	5′‐ACGACCCGAAAGCCTTCATC‐3′
*ribT* qPCR fwd	5′‐CAGCATTTGAGTGTGAACCC‐3′
*ribT* qPCR rev	5′‐CTTGCTGTTTGCTCATTTCC‐3′
*gatB* qPCR fwd	5′‐TCGAGATCGTGTCTGAGC‐3′
*gatB* qPCR rev	5′‐CACAACGCAAGGAACCTT‐3′
*msrA/B* qPCR fwd	5′‐CGACGGGACATTATGAGGCA‐3′
*msrA/B* qPCR rev	5′‐ATTGCCCGCCTACATCAGTC‐3′
*msrA* qPCR fwd	5′‐GCTATGCAGGTGGTCATGTA‐3′
*msrA* qPCR rev	5′‐CGCCATCATCAGTTGGATCG‐3′
*msrA/B* IM fwd	5′‐ATGCAAGCTTCTAGGCCAACCACATCCACTA‐3′
*msrA/B* IM rev	5′‐GCATGGATCCTTCAAAGCCGATTGCGACA‐3′
A/B_F	5′‐GCTACATTTGCTGGAGGATGC‐3′
phY_R	5′‐ACGACTCACTATAGGGCGAATTGG‐3′
A/B_R	5′‐CTGGACCGTCTGGAAATACGTG‐3′
*msrA*_F	5′‐CATACGAACTCGCAACCTTCG‐3′
*msrA*_R	5′‐TATAAAGTCTTCACGGCCAG‐3′
*clpX* EV fwd	5′‐ATGCTGGTACCTCGTGTACATACGAAGGCAAGAG‐3′
*clpX* EV rev	5′‐AGCATGAGCTCTCCGCGGAGGAACATAGCTA‐3′
C16S fwd	5′‐TGCTGGAGGAAGCTTCTGGTGTA‐3′
C16S rev	5′‐AATGTAGCTAACTCTACCTTTTCATTTAC‐3′
C291S fwd	5′‐ACTTCGTTATAGCATAAATTCAGCAGCTCTTCG‐3′
C291S rev	5′‐CCATTCGGTCCTGGACCG‐3′

### 2.2 Construction of the *msrA/B* Mutant and Complement Strains

A 342bp internal fragment of the *msrA/B* gene (locus tag: GBAA_RS27695) was amplified with *msrA/B* IM fwd and *msrA/B* IM rev primers containing restriction sites for HindIII and BamHI (Table 1). The resulting amplicon was subcloned into the temperature‐sensitive plasmid pHY304 (Chaffin et al. 2000), transformed into electrocompetent MC1061F *E. coli* cells (Lucigen, Middleton, WI), and selected on Erm500. The plasmid was then passed through the methylation‐deficient *E. coli* strain, GM2163, using Amp100 selection before being transformed into electrocompetent *B. anthracis* as previously described (Koehler et al. 1994). Transformants were initially plated on Erm5 plates at 30°C, then passed twice at 37°C to force plasmid integration before plating at 37°C on Erm5. Integration of the mutagenic plasmid into the chromosome was confirmed by PCR using a chromosome‐specific forward primer (A/B_F) that amplifies upstream of the site of homologous recombination and a plasmid‐specific primer (phY_R) (Table 1). Disruption of *msrA/B* was further confirmed using primers A/B_F and A/B_R that flank the plasmid insertion site (Table 1). To confirm that plasmid integration occurred only within *msrA/B* and did not disrupt the independent *msrA* (locus tag: GBAA_RS09250) gene, primers *msrA*_F and *msrA*_R (Table 1) were used to amplify *msrA*.

To generate the complementation plasmid p*msrA/B*, the wild‐type *msrA/B* gene was synthesized by Azenta Biotechnical Services (South Plainfield, NJ) and cloned into the multiple cloning site of plasmid pUTE657 downstream of an isopropyl‐β‐d‐1‐thiogalactopyranoside (IPTG; IBI Scientific, Peosta, IA) inducible promoter (Pflughoeft et al. 2011). Empty pUTE657 and p*msrA/B* were passed through GM2163, transformed into *B. anthracis* Sterne Δ*msrA/B*, selected on Spec100, and confirmed by PCR using pUTE657 fwd and rev primers (Table 1). To generate the *clpX* complement in the pDCerm expression plasmid (Jeng et al. 2003), *clpX* (locus tag: GBAA_RS22870) was amplified from WT *B. anthracis* Sterne using the *clpX* EV fwd and *clpX* EV rev primers containing sites for the restriction enzymes KpnI and Sac1 (Table 1). The amplicon was then subcloned into the pDCerm plasmid, passed through GM2163, and transformed into Δ*clpX* using Erm5 for selection. As a control, empty pDCerm was also transformed into WT *B. anthracis* and Δ*clpX*.

To mutate the critical cysteine residue found in the catalytic domains of MsrA/B, a substitution mutation at position 16 from cysteine (TGC) to serine (AGC) and at position 291 from cysteine (TGT) to serine (AGC) was made on p*msrA/*B using site‐directed mutagenesis (NEB) following manufacturer's protocol with primers C16S fwd and C16S rev (Table 1) to generate p*msrA/B* ^C16S^ and primers C291S fwd and C291S rev to generate p*msrA/B* ^C291S^. Mutations were confirmed by whole plasmid sequencing (Plasmidsaurus). Plasmids were transformed into Δ*msrA/B* as described for p*msrA/B*.

### 2.3 Antimicrobial Susceptibility

Overnight cultures were diluted to an optical density wavelength of 600 nm (OD600) of ~0.01 and grown to log phase, as determined by OD600 between 0.4 and 0.5. Media type varied by antimicrobial agent to optimize activity. For assays with penicillin, bacitracin, vancomycin, and H_2_O_2,_ log phase cultures were diluted 1:20 in BHI in a final volume of 200 μL and incubated 16–20 h in 96‐well plates under static conditions at 37°C at the following concentrations: 1.25–80 μg/mL penicillin, 100–400 μg/mL bacitracin, 2–6 μg/mL vancomycin and 0.002%–0.016% of H_2_O_2_. For daptomycin, LL‐37, and paraquat assays, log phase cultures were grown in BHI, then were pelleted, washed in phosphate‐buffered saline (PBS) to remove BHI and re‐pelleted. For daptomycin assays, washed and pelleted cultures were resuspended in an equivalent volume of Mueller‐Hinton II broth (Hardy Diagnostics) supplemented with 50 μg/mL calcium (CA‐MHB) and then diluted 1:20 in a final volume of 400 μL CA‐MHB and incubated for 18–22 h in 5‐mL culture tubes at 37°C shaking at 1–16 μg/mL daptomycin. For paraquat and LL‐37 assays, washed and pelleted cultures were resuspended in an equivalent volume of Roswell Park Memorial Institute (RPMI) 1640 media (Corning) supplemented with lysogeny broth (LB) (Hardy Diagnostics) at either 10% LB (paraquat assays) or 5% LB (LL‐37 assays) then diluted 1:100 (paraquat) or 1:10 (LL‐37) in respective media and incubated in a 200 μL final volume in 96‐well plates under static conditions at 37°C for 20–24 h at the following concentrations: 1–4 μM LL‐37 and 1–8 mM paraquat. Following incubation, the OD600 of each well was measured, and the minimum inhibitory concentration (MIC) was recorded. IPTG was used to induce expression in pUTE657 at 0.1 or 0.05 mM for complementation assays with the plasmids p*clpX* and p*msrA/B*, respectively. Each assay was performed at least three times on different days to generate independent biological replicates.

### 2.4 Growth Curves

For growth curves, overnight cultures were diluted to an OD600 of ~0.01 and grown to log phase, as determined by an OD600 between 0.4 and 0.5. Growth curve assays were set up in the same manner as the antimicrobial assays, using the same antimicrobial concentrations and media conditions as previously described, with the exception that the shaking assays were performed in round‐bottom 96‐well plates at a final volume of 200 μL. Plates were incubated in a CLARIOstar Plus plate reader (BMG Labtech, Offenburg, Germany) at 37°C under shaking conditions (300 rpm) for all assays, except paraquat assays, which were grown under static conditions. OD600 readings (20 flashes, 1800‐s cycle length) were taken every hour for 15 h for assays with vancomycin, bacitracin, daptomycin, and H_2_O_2_, for 24 h for paraquat assays, and for 30 h with LL‐37 assays. At each time point, OD600 values were blank‐corrected by subtracting the corresponding media blank. Unless noted otherwise, growth curves were repeated at least three times on separate days to generate independent biological replicates, and the average optical density +/‐ SD was plotted for each time point.

### 2.5 RNA Extraction

RNA used for operon characterization was extracted as previously described (Franks et al. 2014). For all other extractions, overnight cultures were diluted to an OD600 of ~0.01 and grown to log phase (OD600 0.4–0.5) in BHI. Penicillin and H_2_O_2_ treated log phase cultures were then diluted 1:20 into a final volume of 20 mL of BHI and exposed to 0 or 20 μg/mL penicillin or 0% or 0.004% H_2_O_2_ (1.3 mM) for 30 min. Log phase cultures exposed to paraquat were first washed in PBS, pelleted, and resuspended in an equivalent volume of RPMI + 10% LB. The resuspended cultures were then diluted 1:20 into 20 mL of RPMI + 10% LB and exposed to 0 or 30 mM paraquat for 30 min. Following exposure, cultures were pelleted, washed once in PBS, pelleted, and resuspended in 200 μL water. 210 μL of Maxwell homogenization buffer (Promega, Madison, WI) was added, and the solution was transferred to a 2 mL vial containing 0.1 mm glass disruptor beads. Cells were mechanically disrupted using the Fast Prep‐24 (MP Biomedicals) with one pulse of 6 m/s for 45 s. Supernatant was transferred to a new tube and 200 μL of Maxwell lysis buffer was added. 400 μL of the solution was loaded into a Maxwell RSC instrument cartridge, and RNA extraction followed the manufacturer's protocols. A second DNase treatment was performed following the NEB DNase I protocol. A Nanodrop One^C^ Spectrophotometer (Thermo Fisher) was used to confirm the purity of RNA, as defined by an A260/280 ratio between 2.0 and 2.1 and an A260/A230 ratio between 2.0 and 2.2, and the concentrations for extracted samples were between 100 and 220 ng/μL.

### 2.6 RT‐PCR

RNA (500 ng) used for operon characterization was reverse‐transcribed with 2 μL of the 10 μM gene‐specific primer (GSP‐1) that primes from the 3′ end of *msrA/B* (Table 1) following the Ultra Flex cDNA kit two‐phase protocol (Quantabio). To control for genomic contamination, a non‐reversed‐transcribed RNA control was prepared from the same RNA extraction and contained identical reaction components, except for no reverse transcriptase (no RT). The resulting no RT control and cDNA were then PCR‐amplified with primer pair A (Table 1), which is designed to amplify the sequence between the gene immediately upstream of *msrA/B*, GBAA_RS27700, and *msrA/B* itself. To confirm successful generation of cDNA using GSP‐1, the resulting cDNA product was PCR‐amplified with primer pair B (Table 1), designed to amplify an internal sequence of *msrA/B*.

### 2.7 qPCR

RNA (500 ng) used for quantitative polymerase chain reaction (qPCR) analysis was reverse‐transcribed with random oligonucleotide primers with the Ultra Flex cDNA Kit (Quantabio), following the manufacturer's standard protocol. qPCR amplification was performed using 5 μL PerfeCTa SYBR Green Fastmix (Quantabio), 4 μL cDNA diluted 1:2, and 0.5 μL of the indicated 10 μM forward and reverse primers (Table 1) for a final volume of 10 μL and run on the StepOnePlus Real‐Time PCR System (Applied Biosystems). Cycling conditions were one cycle of 95°C for 10 min, followed by 40 cycles of 95°C for 15 s and 60°C for 1 min. Primers were designed to amplify a 100–200 bp segment of each target gene. Per established standards, PCR amplification efficiency for each primer set was determined using standard curves to ensure consistent doubling between cycles, and all primer pairs had efficiencies between 90% and 110%. Melt curve analysis was also performed to confirm amplification specificity, and no template controls were included for each primer pair (Bustin et al. 2025). Unless indicated otherwise, each treatment condition consisted of three independent biological replicates isolated from separate RNA extractions performed on different days. Each biological replicate was analyzed in technical triplicate. Technical replicates were evaluated for consistency, as defined by an SD < 0.5, and at least two technical replicates were used to determine the Ct value for each biological replicate (Bustin et al. 2025). Individual reactions with Ct values > 30 were considered below the detection threshold and were excluded per guidelines outlined in MIQE 2.0 (Bustin et al. 2025). Relative gene expression was calculated using the Livak method (Livak and Schmittgen 2001) and normalized to a reference gene for each treatment group. Reference genes for each treatment condition were selected due to their stability across treated and untreated samples. The following references genes were used: *ABC‐transporter* (locus tag: GBAA_RS27655), for untreated WT and Δ*clpX* comparisons and for untreated Δ*clpX* and penicillin‐treated Δ*clpX* comparisons; *16S rRNA* for untreated WT and penicillin‐treated WT comparisons, *ribT* (locus tag: GBAA_RS20790) for untreated WT and hydrogen peroxide‐treated WT comparisons; and *gatB* (locus tag: GBAA_RS01810) for untreated WT and WT paraquat‐treated comparisons.

### 2.8 Bioinformatics and Computational Modeling of MsrA/B Mutants

The protein sequence of *B. anthracis* MsrA/B (UniProt: A0A6L8NXQ5) was obtained from the genome annotation of the *B. anthracis* Ames ancestor, which is identical to the corresponding sequence in *B. anthracis* Sterne. Multiple sequence alignments were performed using Clustal Omega with *Bacillus subtilis* MsrA (UniProt: P54154), *Streptococcus pneumoniae* MsrA/B 1 (UniProt: P0A3Q9), *Helicobacter pylori* MsrA/B (UniProt: O25011), and *Haemophilus influenzae* MsrA/B (UniProt: P45213). Pairwise sequence identities between *B. anthracis* MsrA/B and MsrA/B proteins from other species, including *cereus* MsrA/B (UniProt: Q814J4), were determined using NCBI BlastP. To assess conservation of the individual catalytic domains, the *B. anthracis* MsrA/B sequence was additionally compared to *B. subtilis* MsrA (UniProt: P54154) and MsrB (UniProt: P54155), as well as the *Escherichia coli* MsrA (UniProt: P0A744) and MsrB (UniProt: P0A746) using pairwise BlastP.

Structural alignments were performed using UCSF ChimeraX (Meng et al. 2023) to assess conservation of protein structure among fused and independent Msr proteins. Structural similarity was quantified using root mean square deviation (RMSD) values (Table 2). Comparisons of Msr proteins were performed using experimentally determined structures and computationally predicted models. Experimentally determined structures were obtained from the Protein Data Bank and included *B. subtilis* MsrB (PDBID: 2KZN), *S. pneumoniae* MsrA/B1 (PDBID: 3E0M), *E. coli* MsrA (PDBID: 2IEM) and MsrB (PDBID: 6SYM), and *H. pylori* MsrA/B (PDBID: 7E43). Experimentally determined structures were unavailable for *B. anthracis* MsrA/B (UniProt: A0A6L8NXQ5), *B. subtilis* MsrA (UniProt: P54154), *S. pneumoniae* MsrA/B2 (UniProt: A0ACD7PQM9), and *H. influenzae* MsrA/B (UniProt: P45213). The amino acid sequences used for structural modeling with AlphaFold 3.0 (Abramson et al. 2024) were identical to those used for the multiple sequence alignment and pairwise identity comparisons. To evaluate the effect of catalytic cysteine mutations on protein folding, structural models of the *msrA/B* ^C16S^ and *msrA/B* ^C291S^ mutants were generated using both AlphaFold 3.0 and ESMFold (Lin et al. 2023) and compared to the wild‐type *B. anthracis* MsrA/B structure.

**Table 2:** Similarity between MsrA/B of *B. anthracis* modeled using AlphaFold 3.0 compared to homologous proteins from indicated species shown using root mean squared deviation (RMSD).

Protein for comparison:	Homologous structure generated with:	All residue RMSD:	A domain RMSD (# of matched residues):	B domain RMSD (# of matched residues):
*S. pneumoniae* MsrA/B2	AlphaFold 3.0	18.17	1.07 (142)	0.55 (129)
*S. pneumoniae* MsrA/B1	X‐ray crystallography	11.53	1.09 (134)	0.68 (126)
*H. influenzae* MsrA/B	AlphaFold 3.0	21.59	1.08 (133)	0.600 (135)
*H. pylori* MsrA/B	X‐ray crystallography	20.50	1.05 (136)	0.65 (134)
*B. subtilis* MsrA	AlphaFold 3.0	0.73	0.40 (171)	
*B. subtilis* MsrB	NMR	5.99		1.95 (95)
*E. coli* MsrA	NMR	3.56	1.50 (151)	
*E. coli* MsrB	X‐ray crystallography	3.08		0.88 (125)

Computational prediction of protein localization was performed using PSORTb version 3.0 (Yu et al. 2010) and SignalP 6.0 (Teufel et al. 2022). PSORTb was used to evaluate potential localization within Gram‐positive bacterial cellular compartments, while SignalP 6.0 was used to assess the presence of N‐terminal signal peptides and predict protein localization. Predictions were based on output scores and localization probabilities generated by each program.

### 2.9 Statistics

All statistical analyses were performed using GraphPad Prism software. All data sets were assessed for normality with the Shapiro‐Wilk test. Statistical significance for multiple comparisons was determined using Welch's one‐way ANOVA, followed by Dunnett's T3 post hoc test. Relative gene expression data was analyzed using one‐sample Student's *t* tests. Differences were considered statistically significant at *p* < 0.05.

## 3 Results

### 3.1 MsrA/B Protein Alignment and Modeling

**Figure 1:**
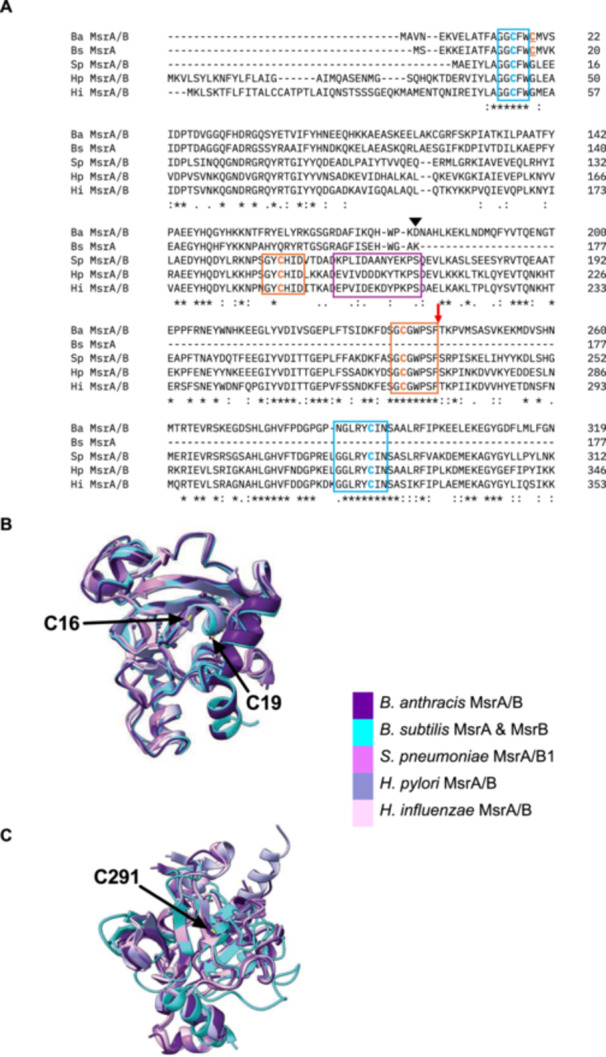
Protein and structural alignment of Msr proteins. (A) Multiple sequence alignment of MsrA/B from *Bacillus anthracis* (Ba), MsrA of *Bacillus subtilis* (Bs), MsrA/B1 of *Streptococcus pneumoniae* (Sp), MsrA/B of *Helicobacter pylori* (Hp), and MsrA/B of *Haemophilus influenzae* (Hi) made using Clustal Omega. Blue and orange boxes indicate catalytic and resolving active sites, respectively. Catalytic cysteines are bold blue, known recycling cysteines are bolded orange, and putative recycling cysteine residues are orange, bolded, and underlined. Established *iloop* sequences are boxed in purple. Red arrow indicates the site of plasmid insertion of the Δ*msrA/B* mutant, and black arrowhead shows the end of MsrA in Bacillus. (*) indicates conserved amino acid, (:) similar amino acid, and (.) semi‐conserved amino acid. Structural alignments of the MsrA (B) and MsrB (C) domains show structural conservation. Structural models were generated of *B. anthracis* MsrA/B, *B. subtilis* MsrA, and *H. influenzae* MsrA/B using AlphaFold 3.0, while experimentally determined structures were used for *B. subtilis* MsrB (PDBID: 2KZN), *S. pneumoniae* MsrA/B1 (PDBID: 3e0m), and *H. pylori* MsrA/B (PDBID: 7e43). The sulfur of the active site cysteine sidechains are colored yellow.

*B. anthracis* contains an *msrA* gene and a fused *msrA/B* gene, which have been previously annotated as *msrA1* and *msrA2*, respectively (Barendt et al. 2013; Pohl et al. 2011). Amino acid sequence alignment analysis showed that the *B. anthracis* MsrA/B shares 40.4% identity with *S. pneumoniae* MsrA/B1, 43.7% with *H. pylori* MsrA/B, and 44.6% identity with *H. influenzae* MsrA/B. Although the overall sequence identity between *B. anthracis* MsrA/B and other fused MsrA/B proteins is relatively low, the catalytic MsrA and MsrB sites (blue box, Figure 1A) and critical cysteine residues (blue bolded C, Figure 1A) are highly conserved. The MsrB recycling sites and respective cysteines are also highly conserved (orange box and orange bolded C, Figure 1A). Structural comparison of the full‐length proteins using AlphaFold 3.0 models and experimentally determined structures yielded high RMSD values (Table 2), indicating differences in domain orientation among species. However, separate alignment of the MsrA (Figure 1B) and MsrB (Figure 1C) domains showed substantial overlap of the conserved catalytic motifs and lower RMSD values (Table 2), indicating that the individual domains retain highly similar structural motifs and architecture.

Comparison of the individual MsrA/B domains with independent Msr enzymes from *B. subtilis* and *E. coli* demonstrates that the MsrB domain is more conserved than the MsrA domain. The *B. anthracis* MsrA domain shares 65.5% amino acid identity with *B. subtilis* MsrA and 40.4% with *E. coli* MsrA, whereas the *B. anthracis* MsrB domain shares 75.6% with *B. subtilis* MsrB and 53.6% identity with *E. coli* MsrB. In contrast to the conserved MsrB recycling site, the putative MsrA recycling site of the *B. anthracis* MsrA/B resembles the *B. subtilis* MsrA, in that there is no separate recycling site. It is hypothesized that the cysteine found immediately after the catalytic site could serve as the recycling cysteine (orange bolded and underlined cysteine in Figure 1A and labeled “C19” in Figure 1B), although this has not been experimentally validated (Boschi‐Muller et al. 2005). The MsrA and MsrB domains of the fused proteins are connected by a short linker sequence, referred to as the *iloop* (Kim et al. 2009). While this region is conserved among other fused MsrA/B proteins (purple box, Figure 1A), this sequence differs in *B. anthracis*. Fused MsrA/B proteins can also differ in their enzymatic domains. Some fused proteins possess a third thioredoxin domain, which functions as a reducing partner for the MsrA and MsrB domains (Lowther et al. 2002; Wu et al. 2005; McGregor and Wolthers 2025). The presence of a third domain has been well studied in *Neisseria* spp. (Lowther et al. 2002; Wu et al. 2005) and more recently reported for the MsrA/B1 of *F. nucleatum* (McGregor and Wolthers 2025). However, this third thioredoxin domain is not universal among fused MsrA/B proteins and is absent in *B. anthracis* MsrA/B.

While fused MsrA/B enzymes from multiple bacterial species have been shown to associate with the cell membrane or cell surface (Skaar et al. 2002; Alamuri and Maier 2004; Saleh et al. 2013; Nasreen et al. 2020), others function intracellularly (Saleh et al. 2013; McGregor and Wolthers 2025). Computational analysis of the *B. anthracis* MsrA/B using PSORTb did not identify a signal peptide, transmembrane helix, or a localization motif. Similarly, SignalP 6.0 predicted a predominantly cytoplasmic localization, with an assigned probability of 0.92 compared to 0.08 for extracellular localization. These findings suggest that *B. anthracis* MsrA/B functions intracellularly. Consistent with this prediction, the fused MsrA/B of *B. cereus*, which shares 96.3% identity with the MsrA/B of *B. anthracis*, has been shown to function intracellularly (Madeira et al. 2017).

### 3.2 Expression of *msrA/B* in *B. anthracis* Sterne

**Figure 2:**
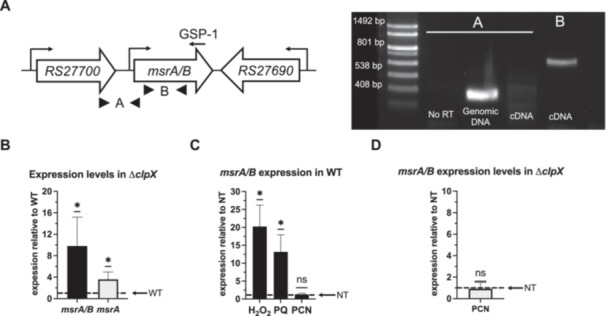
Expression of *msrA/B* in *B. anthracis*. (A) Genomic organization near *msrA/B* gene with the immediate upstream (RS27700; AI‐2E family transporter) and downstream (RS27690; RND efflux transporter) genes. Arrowheads indicate primer pairs A and B; the arrow denotes the gene‐specific primer (GSP‐1) used for reverse transcription; bent arrows indicate putative promoters. Representative PCR generated with primer pairs A or B and the following templates: no reverse transcriptase (RT) control, genomic DNA, or cDNA generated using GSP‐1. Ladder size is indicated on the left. (B) Relative expression of *msrA/B* and *msrA* in Δ*clpX*. Wild‐type (WT) expression levels indicated by dotted line and arrow. (C) *msrA/B* expression in WT following treatments with H_2_O_2_, paraquat (PQ) or penicillin (PCN). Non‐treated (NT) expression levels indicated by dotted line and arrow. (D) *msrA/B* expression in Δ*clpX* treated with PCN. Non‐treated (NT) expression levels indicated by dotted line and arrow. Data is represented as mean ± SD of relative expression of at least three independent RNA extractions, except for PCN‐treated Δ*clpX* which represents 2 independent RNA extractions. * indicates a *p*‐value < 0.05 and ns indicates non‐significance, as determined by a one‐sample *t*‐test.

In *B. anthracis, msrA* and *msrA/B* are in different regions of the genome and have independent regulatory mechanisms. Immediately upstream of *msrA/B* is a gene (locus tag: GBAA_RS27700), putatively annotated as an AI‐2E family transporter, and immediately downstream is a gene (locus tag: GBAA_RS27690) putatively annotated as an RND efflux transporter (left panel, Figure 2A). RS27690 is located on the complementary DNA strand and would not be regulated with *msrA/B*, but RS27700 is located immediately upstream in the same orientation. To determine if *msrA/B* and RS27700 are regulated together as an operon, which could imply a functional connection, we used an RT‐PCR approach (Gupta 1999) to determine if they are transcribed together. RNA was collected from wild‐type cells and reverse‐transcribed with the gene‐specific primer GSP‐1 (arrow; left panel, Figure 2A). The resulting cDNA was then amplified with two primer sets: primer pair A, whose forward and reverse primers span the sequence between RS27700 and *msrA/B*, and primer pair B, which amplifies an internal portion of *msrA/B* (arrowheads; left panel, Figure 2A). No amplification was seen in the no‐reverse‐transcriptase (No RT) control, and as expected, amplification was seen with primer pair A using genomic DNA (right panel, Figure 2A). Primer pair B amplified a band with cDNA generated using GSP‐1, demonstrating that reverse transcription was successful, however, amplification of cDNA with primer pair A did not result in a band (right panel, Figure 2A). This indicates that *msrA/B* and RS27700 are not co‐transcribed and are less likely to be functionally connected. Our findings mirror similar conclusions with the *B. cereus msrA/B*, which was found to be transcribed as an independent unit (Madeira et al. 2017).

Previously, we found that *msrA/B* was overexpressed in Δ*clpX* in our microarray (Claunch et al. 2018). We confirmed this with qPCR and found a ~9.8‐fold increase in *msrA/B* levels in Δ*clpX* as compared to WT (Figure 2B). Although *msrA* was not identified in the microarray as dysregulated in Δ*clpX*, we also assessed its expression by qPCR and found that expression of *msrA* is also significantly higher in Δ*clpX* as compared to WT (~3.6‐fold), although to a lesser extent than *msrA/B* (Figure 2B). Next, we tested H_2_O_2_ and paraquat, oxidants known to induce *msrA/B* in other species (Alamuri and Maier 2006; Sasindran et al. 2007; Singh et al. 2018), and found that *msrA/B* expression was significantly increased in WT by H_2_O_2_ (~20‐fold increase) and by paraquat (~13‐fold increase), as compared to untreated WT samples (Figure 2C). Finally, we investigated whether penicillin, a beta‐lactam antibiotic, would alter expression of *msrA/B* in *B. anthracis*, as was seen with cell wall antibiotics in *S. aureus* (Wilkinson et al. 2001; Utaida et al. 2003). However, penicillin had no effect on *msrA/B* expression in wild‐type (Figure 2C), nor Δ*clpX* (Figure 2D). Thus, in *B. anthracis* Sterne, *msrA/B* expression is induced by the oxidants H_2_O_2_ and paraquat, but not by penicillin.

**Figure 3:**
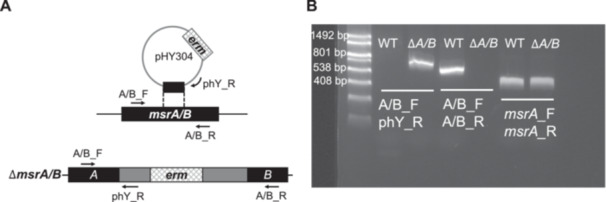
Insertional mutagenesis of *msrA/B*. (A) Diagram depicting the generation of the insertional mutant Δ*msrA/B*, with the wild‐type *msrA/B* gene (black), the mutagenesis plasmid pHY304 (gray), and the erythromycin‐resistance gene (*erm*; crossed pattern). Primers used for mutant confirmation are indicated with black arrows. (B) Representative gel of PCR products from wild‐type (WT) *B. anthracis* Sterne and the Δ*msrA/B* (Δ*A/B*) mutant amplified with indicated primer pairs.

To test the functional contributions of *msrA/B*, we generated the Δ*msrA/B* mutant by insertional mutagenesis using homologous recombination of the pHY304 plasmid between the recycling and catalytic site of the MsrB domain (red arrow, Figures 1A and 3A). Insertion of the plasmid into *msrA/B* was confirmed by PCR, using the forward primer A/B_F, designed to anneal to the genomic sequence upstream of the insertion site (Figure 3A), and a reverse primer, phY_R, designed to anneal to the pHY304 plasmid. No amplification was seen with WT *B. anthracis* using this primer set, but amplification in the Δ*msrA/B* mutant confirmed plasmid insertion (Figure 3B). For further confirmation, a second primer set consisting of the same forward primer A/B_F and A/B_R, a reverse primer that primes downstream of the insertion site (Figure 3A), was used with genomic DNA from WT or Δ*msrA/B*. As expected, amplification with WT genomic DNA was successful, but not with Δ*msrA/B* that contains the integrated plasmid (Figure 3B). To ensure there was no unintentional disruption of the independent *msrA* gene, we used *msrA*‐specific primers (*msrA*_F and *msrA*_R) to amplify genomic DNA from WT or *msrA/B*, producing equal‐sized bands from both strains, which indicates no plasmid insertion (Figure 3B).

### 3.3 ClpX But Not MsrA/B Contributes to Oxidative Stress Resistance

**Figure 4:**
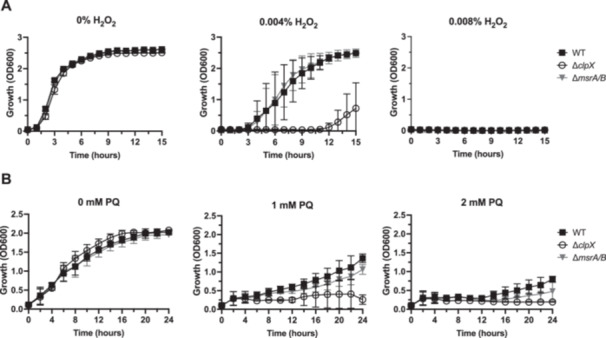
Growth of Δ*clpX* is inhibited in ROS. Growth of wild‐type (WT) *B. anthracis* Sterne, Δ*clpX*, and Δ*msrA/B* in (A) BHI containing indicated levels of hydrogen peroxide (H_2_O_2_) and (B) RPMI + 10% LB containing paraquat. The absorbance (OD600) was measured every hour for 15–24 h at 37°C shaking (H_2_O_2_) or static (paraquat) and data is represented as mean ± SD of three independent biological replicates, except for the 0.008% H_2_O_2_ assay which has two independent biological replicates.

It has been well established that Msr‐deletion mutants are more susceptible to oxidative stress, though the susceptibility to specific types of ROS varies (Ezraty et al. 2005; Sasindran et al. 2007; Singh et al. 2018). ClpX has also been linked to oxidative stress in several bacterial species, including *Glaesserella parasuis, Streptococcus suis*, *E. coli*, and *S. aureus* (Xu et al. 2025; Roy et al. 2019; Sen et al. 2020; Frees et al. 2003), although its importance has not been established in *B. anthracis*. ROS sensitivity of Δ*clpX* and Δ*msrA/B* was determined with MIC assays using H_2_O_2_ and paraquat. We found that Δ*clpX* had a lower MIC for both oxidants, whereas Δ*msrA/B* showed no change in MIC (Table 3). We next used growth curves to assess whether the growth of Δ*clpX* or Δ*msrA/B* was delayed in the presence of either H_2_O_2_ or paraquat. As expected, the growth of Δ*clpX* was inhibited as compared to WT (Figure 4). In contrast, Δ*msrA/B* had no growth defects in H_2_O_2_ but exhibited a minor growth delay in paraquat (Figure 4).

**Table 3:** MICs of wild‐type (WT) *B. anthracis* Sterne and mutants.

Strains	H_2_O_2_ (%)	Paraquat (mM)
WT	0.016	4
Δ*clpX*	0.008	1
Δ*msrA/B*	0.016	4

**Figure 5:**
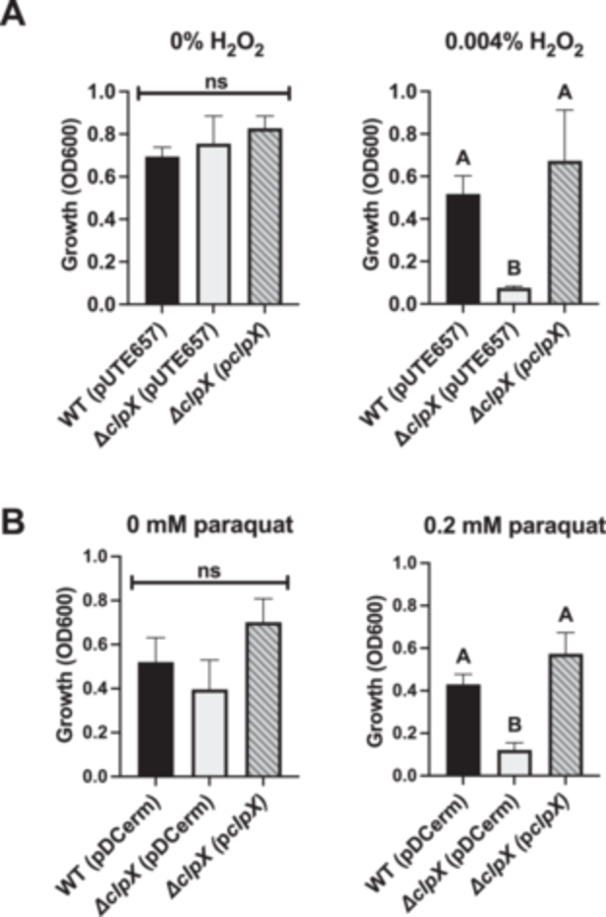
Complementation with p*clpX* restores resistance to ROS. (A) Overnight growth of wild‐type (WT) or Δ*clpX B. anthracis* Sterne containing the empty plasmid (pUTE657) or expressing the *clpX* gene (p*clpX*) in BHI at indicated concentrations of H_2_O_2_. (B) Growth of WT *B. anthracis* or Δ*clpX B. anthracis* containing the empty plasmid (pDCerm) or expressing the *clpX* gene (p*clpX*) in RPMI + 10% LB at indicated concentrations of paraquat (PQ). Data is represented as mean ± SD of at least four independent biological replicates. Statistically significant differences (*p*‐value < 0.05) are represented by different letters, and ns indicates non‐significance, as determined by Welch's ANOVA, followed by Dunnett's T3 post hoc test.

To confirm that the H_2_O_2_ sensitivity of Δ*clpX* is due to loss of *clpX*, we complemented the Δ*clpX* mutant with the *clpX* gene expressed on the IPTG‐inducible plasmid pUTE657. Empty pUTE657 was included in WT *B. anthracis* Sterne and Δ*clpX* to control for any plasmid effect. There was no difference in overnight growth between any of the strains in the absence of H_2_O_2_ (left panel, Figure 5A). With H_2_O_2_ exposure, WT containing pUTE657, and Δ*clpX* complemented with p*clpX* showed no difference in growth after overnight incubation, while Δ*clpX* with pUTE657 maintained its increased sensitivity (right panel, Figure 5A). We next performed a similar complementation assay with paraquat; however, we found a strong plasmid effect with pUTE657 in the WT and Δ*clpX* strains in the presence of paraquat whereby growth of even the wild‐type bacteria was severely impacted thus mitigating any phenotypic difference between strains. To compensate, we used a different expression plasmid, pDCerm. While the pDCerm plasmid did decrease the MIC for all strains carrying it, the difference in growth between WT and Δ*clpX* in the presence of paraquat was maintained (right panel, Figure 5B), and there was no difference in growth between strains in the absence of paraquat (left panel, Figure 5B). Expression of *clpX* from pDCerm complemented Δ*clpX* and restored WT‐levels of growth in paraquat (right panel, Figure 5B). We conclude that ClpX is required for resistance to H_2_O_2_ and paraquat, but MsrA/B is not.

### 3.4 Loss of MsrA/B Increases *B. anthracis* Sensitivity to Penicillin

**Figure 6:**
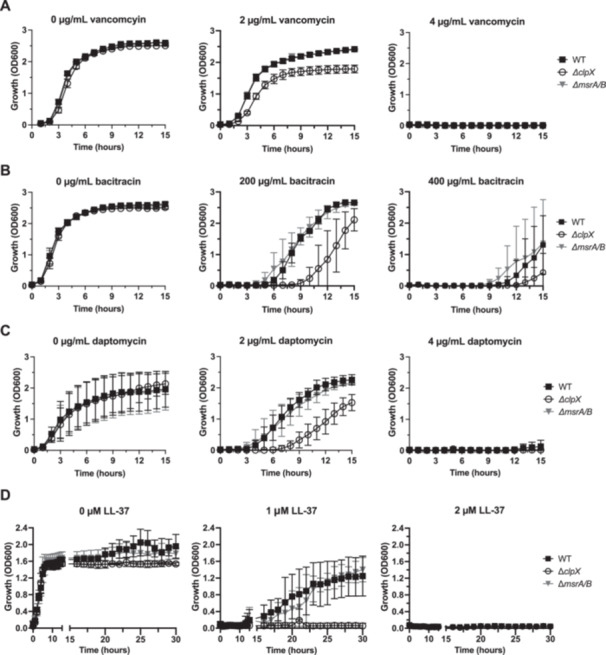
Growth of Δ*clpX* is inhibited by cell envelope targeting antimicrobials. Growth of wild‐type (WT) *B. anthracis* Sterne, Δ*clpX*, and Δ*msrA/B* in (A) BHI containing vancomycin (B) BHI containing bacitracin (C) CA‐MHB containing daptomycin or (D) RPMI + 5% LB containing LL‐37 at indicated concentrations. The absorbance (OD600) was measured every hour for 15 h for vancomycin, bacitracin and daptomycin assays and 30 h for LL‐37 assays at 37°C shaking. Data is shown as mean ± SD of three independent biological replicates, except for 400 μg/mL bacitracin which has two independent biological replicates.

Initially, we hypothesized that loss of *msrA/B* would increase resistance to cell wall antibiotics. This was based on findings in *S. aureus* that loss of *msrB* increased resistance to oxacillin (Singh et al. 2015) and the fact that the Δ*clpX* mutant, which is more sensitive to cell wall antibiotics, overexpresses *msrA/B*. To test this hypothesis, we conducted MIC assays with WT, Δ*clpX*, and Δ*msrA/B* and, as previously shown, Δ*clpX* is more susceptible to the cell envelope targeting antibiotics penicillin, vancomycin, bacitracin, and daptomycin, as well as LL‐37, a human antimicrobial peptide (McGillivray et al. 2012; Zou et al. 2021) (Table 4). However, contrary to our hypothesis, loss of *msrA/B* resulted in increased sensitivity to penicillin (Table 4). There was no difference in the MIC of WT and Δ*msrA/B* with the other tested cell wall targeting antibiotics, vancomycin and bacitracin, nor with the cell membrane targeting antibiotics daptomycin and LL‐37 (Table 4). To ensure there was no growth delay in the Δ*msrA/B* mutant, we performed growth curves with these antibiotics. We saw no differences in growth between WT and Δ*msrA/B* with either cell wall or cell envelope (Figure 6A–D) targeting antibiotics, although growth of Δ*clpX* was inhibited by all of them.

**Table 4:** MICs of wild‐type (WT) *B. anthracis* Sterne and mutants.

Strains	Penicillin (μg/mL)	Vancomycin (μg/mL)	Bacitracin (μg/mL)	Daptomycin (μg/mL)	LL‐37 (μM)
WT	80	4	200	16	2
Δ*clpX*	1.25	2	100	8	1
Δ*msrA/B*	20	4	200	16	2

**Figure 7:**
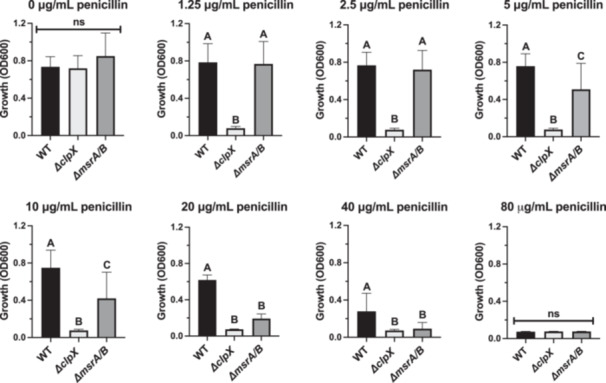
Loss of *msrA/B* increases sensitivity to penicillin. Growth of wild‐type (WT) *B. anthracis* Sterne, Δ*clpX*, and Δ*msrA/B* in BHI containing indicated concentrations of penicillin. Data is represented as mean ± SD of at least three independent biological replicates. Statistically significant differences are represented by different letters (*p*‐value < 0.05) and ns indicates non‐significance, as determined by Welch's ANOVA, followed by Dunnett's T3 post hoc test.

Despite Δ*clpX* and Δ*msrA/B* both having increased sensitivity to penicillin, Δ*clpX* is markedly more sensitive than Δ*msrA/B*, with full inhibition of Δ*clpX* overnight growth occurring at 1.25 μg/mL (Table 4 and Figure 7). In contrast, while a partial inhibition of growth is seen with Δ*msrA/B* at 5 μg/mL, full growth inhibition is not seen consistently until 20 μg/mL (Figure 7). This suggests that while MsrA/B plays a role in the penicillin resistance of *B. anthracis* Sterne, it is to a lesser extent than ClpX.

### 3.5 Enzymatic Activity of MsrA/B Is Required for Resistance to Penicillin

**Figure 8:**
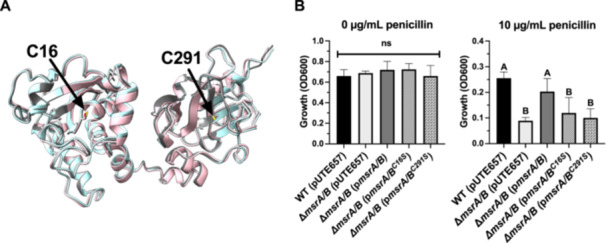
Enzymatic activity of MsrA/B is required for resistance to penicillin in *B. anthracis* Sterne. (A) Structural models were generated using AlphaFold 3.0 for MsrA/B (gray), C16S (pink), and C291S (blue). The sulfur of the cysteine sidechain is colored yellow, while the oxygen of serine sidechain is colored red. (B) Overnight growth of WT or Δ*msrA/B* containing empty plasmid (pUTE657) or expressing the wildtype *msrA/B* gene (p*msrA/B*) or cysteine mutants (p*msrA/B* ^*C16S*^ or p*msrA/B* ^*C291S*^) in BHI containing the indicated amount of penicillin. Data is represented as mean ± SD of four independent biological replicates. Statistically significant differences (*p*‐value < 0.05) are represented by different letters and ns indicates non‐significance, as determined by Welch's ANOVA, followed by Dunnett's T3 post hoc test.

Finally, we assessed if the enzymatic activity of MsrA/B is required for penicillin resistance. We first complemented Δ*msrA/B* with the wild‐type *msrA/B* gene to generate p*msrA/B*. Next, we mutated the catalytic cysteines of the MsrA‐portion (C16) and the MsrB‐portion (C291) to serine to create the p*msrA/B* ^C16S^ and p*msrA/B* ^C291S^ plasmids. Swapping the single atom in the thiol of the catalytic cysteine to the hydroxyl of serine has previously been shown to abolish Msr reducing activity while preserving the overall protein structure (Moskovitz et al. 2000; Boschi‐Muller et al. 2000; Olry et al. 2002; Kim et al. 2021). To assess whether these substitutions are predicted to alter MsrA/B structure in *B. anthracis*, we modeled both mutants using AlphaFold 3.0 (Figure 8A) and ESMFold. Both approaches predicted structures highly similar to wild‐type MsrA/B, with backbone RMSD values of 0.04 Å (ESMFold) and 0.72 Å (AlphaFold 3.0) for the C16S mutant, and 0.16 Å (ESMFold) and 0.49 Å (AlphaFold 3.0) for the C291S mutant, consistent with minimal structural disruption seen in other Msr proteins (Moskovitz et al. 2000; Kim et al. 2021). Empty pUTE657 was included in WT and Δ*msrA/B* to control for any plasmid effects. There was no difference in overnight growth in the absence of penicillin among the strains (left panel, Figure 8B). In the presence of penicillin, we found that p*msrA/B* restored WT levels of resistance to Δ*msrA/B*, confirming that MsrA/B contributes to penicillin resistance. However, p*msrA/B* ^C16S^ and p*msrA/B* ^C291S^ were unable to complement Δ*msrA/B* and there was no difference in growth between Δ*msrA/B* (pUTE657) and Δ*msrA/B* (p*msrA/B* ^C16S^) or Δ*msrA/B* (p*msrA/B* ^C291S^) (right panel, Figure 8B), indicating that both catalytic cysteines are essential for this phenotype. We conclude that the reducing activity of MsrA/B is essential for *B. anthracis* Sterne resistance to penicillin. Further, both the MsrA and MsrB enzymatic domains are required for this function, as the presence of one catalytic site cannot compensate for the loss of the other.

## 4 Discussion

Given the well‐established role of Msr enzymes in the bacterial oxidative stress response, and the strong induction of *msrA/B* with H_2_O_2_ and paraquat (Figure 2C), it was unexpected to find that loss of MsrA/B does not alter ROS resistance in *B. anthracis* Sterne (Table 3). Although we did not experimentally validate the reducing capability of the *B. anthracis* MsrA/B enzyme, studies of the highly related *B. cereus* MsrA/B protein (96.3% identity) demonstrate that deletion of *msrA/B* alters the methionine oxidation state of the proteome, with complementation restoring MetO levels observed in wild‐type cells (Madeira et al. 2017). Additionally, both Msr domains and active sites are highly conserved (Figure 1A and Table 2) with species whose Msr enzymes have been shown to reduce methionine sulfoxide (Kim et al. 2009; Boschi‐Muller et al. 2005; Kim et al. 2021; Nasreen et al. 2022), providing strong evidence that this enzyme functions in the predicted manner. Our expression data is also consistent with findings in *B. anthracis* strain USM23C‐1, where *msrA/B* (annotated as *msrA‐2*) was induced by H_2_O_2_ and paraquat (Pohl et al. 2011). However, ROS‐induced *msr* expression does not always correspond to ROS susceptibility. An *H. influenzae* Δ*msrA/B* mutant showed no susceptibility to either H_2_O_2_ or paraquat, despite both inducing *msrA/B* expression (Nasreen et al. 2020). The opposite response is seen in *S. aureus*, where oxidants do not upregulate any of the four *msr* genes (Singh and Singh 2012) but deletion of *msrA1* increased ROS sensitivity, while loss of *msrB* had no effect (Singh et al. 2015). Thus, there is not always a direct connection between the induction of *msr* genes by oxidative stress and sensitivity to that same stressor. Similarly, while Msr‐deficient mutants are often sensitive to H_2_O_2_ and paraquat in multiple species (Ezraty et al. 2005; Sasindran et al. 2007; Singh et al. 2018), this is not universal. As an example, the MsrA/B of *H. influenze* has only been found to be susceptible to hypochlorous acid stress, but not to other oxidants (Nasreen et al. 2020), whereas the MsrA/B of *H. pylori* is sensitive to H_2_O_2_ and paraquat (Alamuri and Maier 2004). Collectively, these findings demonstrate that Msr phenotypes can vary significantly across species and oxidants.

One potential explanation for this variance in phenotypes between Msr mutants is the unequal contribution of MsrA and MsrB to ROS resistance. In several species, including *S. typhimurium*, *Mycobacterium smegmatis*, and *S. aureus*, deletion of MsrA yields a more severe ROS‐sensitive phenotype than the loss of MsrB (Denkel et al. 2011; Dhandayuthapani et al. 2009; Singh et al. 2015). Thus, it is possible that the independent MsrA in *B. anthracis* may play a more significant role in the ROS response and can compensate for the loss of MsrA/B. Additionally, *B. anthracis* has a robust oxidative stress response, consisting of multiple catalases, peroxidases, superoxide dismutases (SODs), and other antioxidant proteins (Tu et al. 2012). Functional redundancy within the ROS response may compensate for disruption of *msrA/B* with the ROS tested here. Finally, it is possible that the lack of altered ROS sensitivity in the Δ*msrA/B* mutant is due to residual activity of one or both Msr domains, as the strain was generated using insertional mutagenesis and those domains remain present in the cell, albeit separated by plasmid insertion. Further experimentation will be needed to investigate these possibilities.

Although we saw no change in ROS susceptibility upon loss of MsrA/B, we did observe decreased resistance with loss of ClpX (Table 3 and Figure 4). This finding is not unique to *B. anthracis*, as it has been reported that loss of ClpX and/or ClpP results in decreased growth and ROS resistance in *Glaesserella parasuis, S. pneumoniae, S. suis*, *E. coli*, and *S. aureus* (Xu et al. 2025; Robertson et al. 2002; Roy et al. 2019; Sen et al. 2020; Frees et al. 2003), but it is the first confirmation that ClpX is also important in mitigating oxidative stress in *B. anthracis* Sterne. We find that Δ*clpX* is sensitive to both hydrogen peroxide and paraquat, which generate different types of oxidative damage, suggesting that ClpX contributes broadly to oxidative stress in *B. anthracis*.

Multiple studies have demonstrated that loss of *clpX* or *clpP* impacts the bacterial stress response (Frees et al. 2007; Illigmann et al. 2021; Aljghami et al. 2022), and some phenotypes associated with Δ*clpX* and Δ*clpP* mutants are likely due to an accumulation of damaged proteins the cell is unable to degrade. However, loss of *clpX* and/or *clpP* also results in the accumulation of specific proteins, such as transcriptional regulators (Feng et al. 2013; Farrand et al. 2015), which can impact gene expression (Frees et al. 2007; Claunch et al. 2018; Aljghami et al. 2022). One such transcriptional regulator is Spx, whose levels are controlled by ClpXP‐mediated degradation in *S. aureus* (Pamp et al. 2006; Feng et al. 2013), and *B. subtilis* (Nakano et al. 2002). Spx induces *msrA/B* in *B. anthracis* (Barendt et al. 2013) and the *msrAB* operon in *B. subtilis* (You et al. 2008). This suggests that the elevated *msrA/B* levels in *B. anthracis* Δ*clpX* may result from Spx accumulation. Consistent with this hypothesis, elevated MsrA1 protein levels were observed in an *S. aureus* Δ*clpX* mutant (Farrand et al. 2015), providing further evidence that loss of ClpX can result in Msr accumulation.

Msr enzymes have primarily been studied in the context of oxidative stress, which is consistent with their established function; however, their potential role in antibiotic resistance remains largely unexplored. Transcriptomic studies of the *S. aureus* cell wall stress response showed that oxacillin, bacitracin, and d‐cycloserine all upregulated the *msrA1B* operon, suggesting a connection between Msr enzymes and antibiotic‐induced stress (Wilkinson et al. 2001; Utaida et al. 2003). Despite this, deletion of *msrA1*, either individually or in a triple knockout with the other two *mrsA* genes found in *S. aureus*, did not alter cell wall antibiotic resistance, while loss of MsrB resulted in increased resistance (Singh et al. 2015). In contrast, we found that *msrA/B* expression was unchanged after penicillin treatment (Figure 2C,D). Surprisingly, our data shows that *msrA/B* disruption increases penicillin sensitivity in *B. anthracis* (Table 4 and Figure 7), despite *msrA/B* levels being elevated in Δ*clpX* (Figure 2B). One possibility for this dichotomy is that MsrA/B levels may require tight regulation, with either excessive or insufficient amounts impairing the antibiotic stress response in *B. anthracis*. However, it is also possible that elevated *msrA/B* levels seen in Δ*clpX* are unrelated to its protective effect against penicillin. Further studies will be required to determine whether MsrA/B levels contribute to penicillin resistance.

Interestingly, disruption of *msrA/B* results in decreased resistance to penicillin, but not to other tested antibiotics that disrupt cell wall synthesis. Although the basis for this selective sensitivity is not fully understood, this may be due to changes in oxidative stress following penicillin treatment. The bactericidal activity of beta‐lactam antibiotics is primarily attributed to the inhibition of transpeptidation of new peptidoglycan units into the existing matrix (Waxman and Strominger 1983). However, some studies have found these antibiotics can also induce oxidative stress as a secondary mechanism of activity (Kohanski et al. 2007; Cho et al. 2014; Van Acker and Coenye 2017; Kawai et al. 2023). Specifically, beta‐lactam‐mediated disruption of cell wall synthesis results in futile cycling of peptidoglycan synthesis and degradation, which increases metabolic demand and promotes ROS accumulation (Cho et al. 2014; Van Acker and Coenye 2017; Léger et al. 2019). This resulting accumulation of ROS may increase the reliance on MsrA/B to repair oxidized proteins during penicillin exposure. In support of this, we found that the reducing activity of both Msr‐domains of MsrA/B are required for penicillin resistance (Figure 8B). There is less evidence linking treatment with other cell wall antibiotics, such as vancomycin or bacitracin, to increases in intracellular oxidative levels (Liu and Imlay 2013; Li et al. 2015; Van Acker and Coenye 2017). This suggests that MsrA/B may be required to reduce intracellular ROS levels caused by altered metabolism during penicillin exposure, rather than disruption of the cell wall.

Regardless, our data indicates that MsrA/B plays only a minor role in the cell envelope antibiotic sensitivity seen with loss of *clpX*. While Δ*clpX* and Δ*msrA/B* share a decreased resistance to penicillin, Δ*clpX* has a stronger phenotype (Figure 7) and is susceptible to other cell envelope antimicrobials (Table 4). This difference in sensitivity is not entirely unexpected, as we have previously shown that loss of *clpX* affects the expression of other genes, such as *lrgAB*, which also contributes to cell envelope antibiotic resistance in *B. anthracis* but to a lesser extent than ClpX (Claunch et al. 2018). This suggests that the ClpXP‐associated cell envelope stress response is mediated through the regulation of multiple downstream targets with distinct functional roles. Further studies will be needed to elucidate the precise mechanisms behind this.

## 5 Conclusions

We find that loss of ClpX leads to upregulation of *msrA/B* expression. Despite this connection, MsrA/B and ClpX have distinct roles in oxidative and cell envelope stress in *B. anthracis*. Although *msrA/B* is induced by hydrogen peroxide and paraquat, ClpX, rather than MsrA/B, is necessary for resistance to these oxidants. MsrA/B does contribute to penicillin resistance, though to a lesser extent than seen with ClpX. Additionally, unlike loss of *clpX*, no change in susceptibility to other cell wall or cell membrane targeting antibiotics is seen with loss of *msrA/B*. Importantly, MsrA/B‐mediated penicillin resistance requires the enzymatic activity of both Msr domains, as mutation of either catalytic site abolishes activity. This suggests that the reducing activity of MsrA/B is necessary for penicillin resistance and that both active sites are required. Our results highlight the complex role of the ClpXP protease in regulating oxidative and cell envelope stress responses.

## Author Contributions

**Aeron B. Pennington:** writing – original draft, funding acquisition, investigation, writing – review and editing, formal analysis, methodology, validation, visualization, conceptualization. **Salina Hona:** investigation, funding acquisition, writing – review and editing, validation, conceptualization. **Josey I. Austin:** investigation, funding acquisition, writing – review and editing, validation. **Kelsey C. Waite:** investigation, funding acquisition, writing – review and editing, validation. **Mikaela D. Stewart:** formal analysis, writing – review and editing, visualization. **Shauna M. McGillivray:** conceptualization, funding acquisition, writing – review and editing, methodology, project administration, supervision, visualization, resources.

## Ethics Statement

The authors have nothing to report.

## Conflicts of Interest

The authors declare no conflicts of interest.

## Data Availability

All data generated or analyzed during this study are included in this published article.
